# Widespread umbilicated papules and nodules in an immunosuppressed patient

**DOI:** 10.1016/j.jdcr.2024.04.007

**Published:** 2024-04-20

**Authors:** Robin Wang, Zisansha Zahirsha, Brandon Zelman, Jodi Speiser, Jenna Lullo

**Affiliations:** a Division of Dermatology, Loyola University Medical Center, Maywood, Illinois; b Department of Pathology and Laboratory Medicine, Loyola University Medical Center, Maywood, Illinois

**Keywords:** blastomycosis, immunosupression, disseminated, systemic mycosis

## Case description

**Fig 1:**
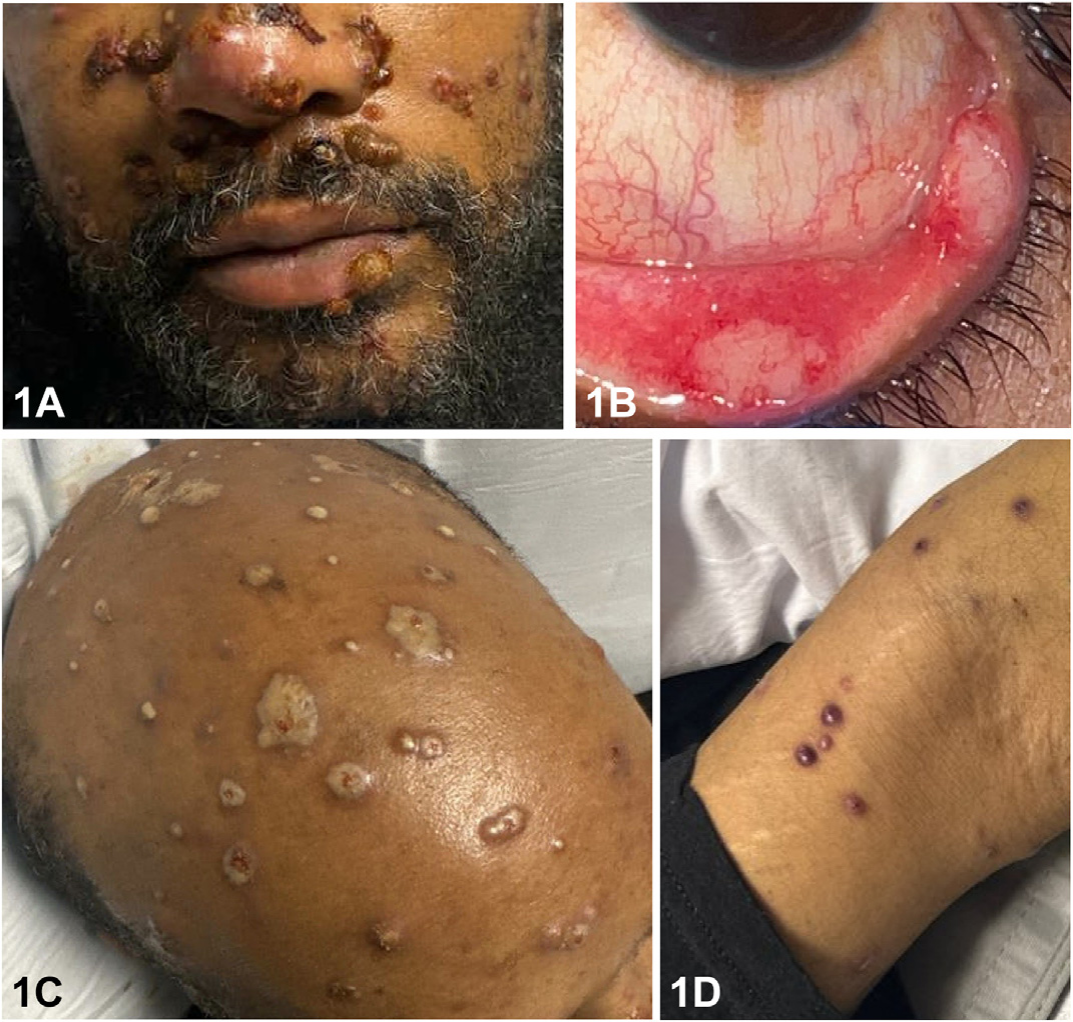

**Fig 2:**
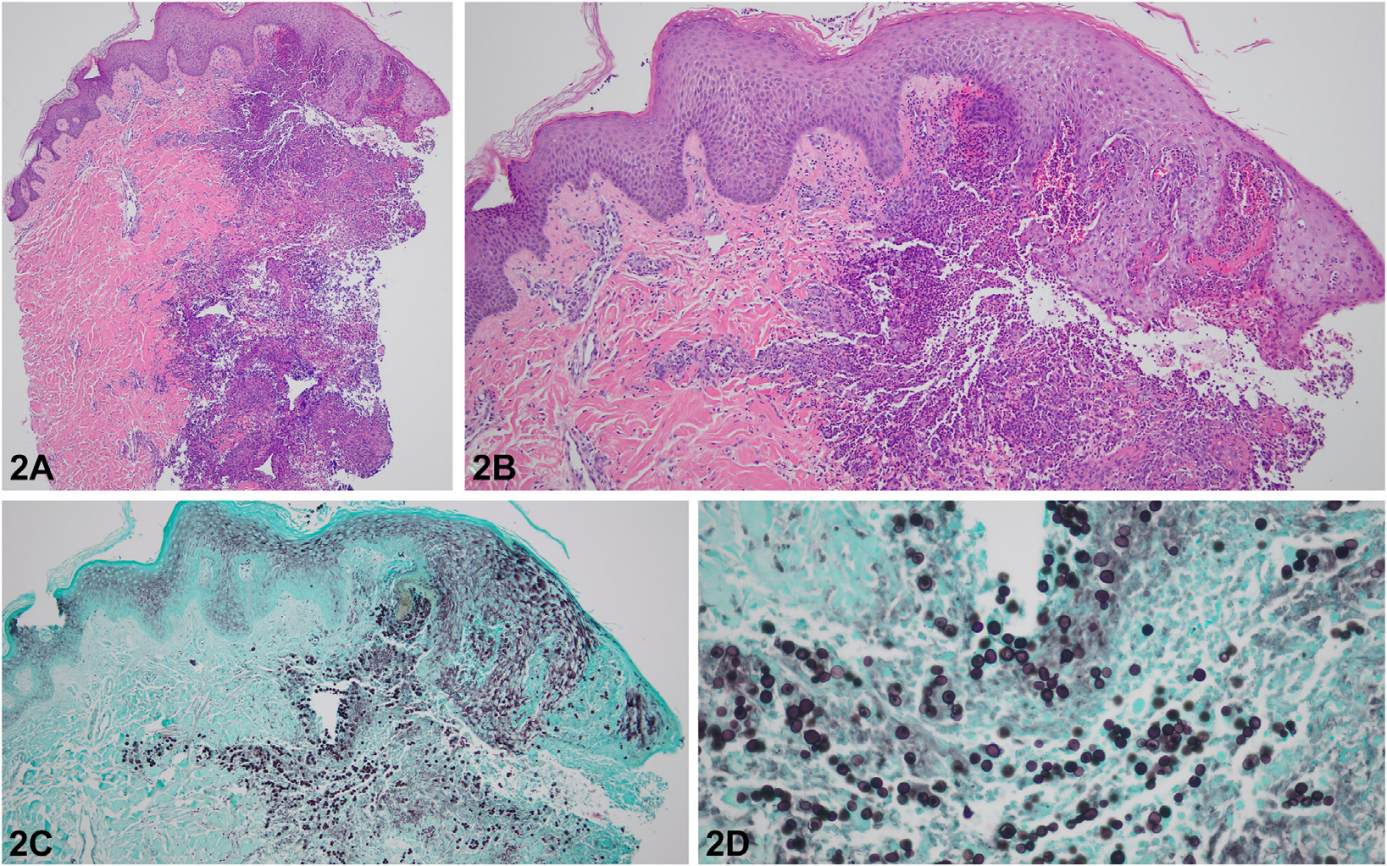

A 45-year-old man from Ohio with end-stage renal disease status postrenal transplantation presented with several weeks of fever, cough, photophobia, and headache. Cutaneous examination was notable for conjunctival erosions, and new painful umbilicated nodules and papules on the face, scalp, and extremities refractory to antibiotics (Fig 1, *A-D*). Radiographic imaging demonstrated multifocal lung opacities. Histopathology findings are demonstrated below (Fig 2, *A-D*).

**Question 1: What is the diagnosis?**

A. Histoplasmosis
B. Blastomycosis
C. Herpes Zoster
D. Monkeypox
E. Coccidiomycosis

**Answers:**

A. Histoplasmosis – Incorrect. Although ulcerative cutaneous nodules and pulmonary disease can be manifestations of disseminated histoplasmosis, histopathology reveals intracellular yeast within histiocytes, not broad-based budding yeast. Of note, there is high cross-reactivity on antigen testing between histoplasmosis and blastomycosis, thus histopathology is key to rapid identification.^1^
B. Blastomycosis – Correct. Disseminated blastomycosis presents with pulmonary disease and cutaneous dissemination is the most common extrapulmonary site. Cutaneous manifestations range from ulcerative papulonodules to verrucous plaques and mucosal ulceration. Central nervous system involvement is more common in immunocompromised hosts and can present with meningitis, epidural abscesses, or intracranial abscesses. Histopathologic examination reveals broad-based budding yeast.^2^
C. Herpes Zoster – Incorrect. Although disseminated herpes zoster can present with a widespread vesicular eruption and pneumonitis in immunocompromised hosts, histopathology reveals viral cytopathic changes including multinucleation, margination of chromatin, and nuclear inclusions.
D. Monkeypox – Incorrect. Although umbilicated pseudo-pustules and nodules and pulmonary disease can be manifestations of disseminated monkeypox in immunocompromised hosts, patients also typically present with proctitis or tonsillitis. In addition, histopathology reveals viral cytopathic changes, not broad-based budding yeast.
E. Coccidiomycosis – Incorrect. Although ulcerative cutaneous nodules and pulmonary disease can be manifestations of disseminated coccidiomycosis, histopathology reveals large spherules containing endospores, not broad-based budding yeast.^3^

**Question 2: What is the geographic distribution of blastomycosis?**

A. Ubiquitous in the environment
B. Worldwide, especially temperate and tropical regions
C. Southwestern United States
D. Central and South America
E. Midwestern, Southeastern, and South-Central United States

**Answers:**

A. Ubiquitous in the environment – Incorrect. Blastomycosis is geographically restricted. Herpesviruses, for example, are ubiquitous in the environment.
B. Worldwide, especially temperate and tropical regions – Incorrect. Sporotrichosis is distributed worldwide, especially in temperate, tropical, and subtropical regions. Infection is acquired through cutaneous inoculation of fungal spores from infected soil.^3^
C. Southwestern United States – Incorrect. Coccidiomycosis is endemic to the Southwest United States, where infection is acquired though inhalation of fungal spores from infected soil.^3^
D. Central and South America – Incorrect. Paracoccidiomycosis is endemic to Central and South America, where infection is acquired though inhalation of fungal spores from infected soil.^3^
E. Midwestern, Southeastern, and South-Central United States – Correct. Blastomycosis is endemic to the Midwestern, Southeastern, and South-Central United States, including the Ohio and Mississippi River Valleys. Infection is acquired through inhalation of fungal spores from infected soil.^1^ Of note, histoplasmosis shares considerable geographic overlap with blastomycosis.^3^

**Question 3: What is the most appropriate initial management?**

A. Itraconazole
B. Amphotericin B
C. Observation
D. Levofloxacin
E. Micafungin

**Answers:**

A. Itraconazole – Incorrect. Oral itraconazole would be appropriate for mild to moderate pulmonary or disseminated blastomycosis without central nervous system involvement in immunocompetent hosts. Serum levels of itraconazole should be assessed after 2 weeks to ensure therapeutic levels.^4^
B. Amphotericin B – Correct. Initial therapy with amphotericin B is necessary in severe pulmonary or disseminated blastomycosis, central nervous system disease, or immunocompromised hosts. Once clinical improvement is realized, therapy may be transitioned to an oral azole.^4^
C. Observation – Incorrect. In cases of acute pulmonary blastomycosis in immunocompetent hosts, disease may be mild, self-limited, and not require treatment. Cases involving immunocompromised hosts and disseminated infection require anti-fungal therapy.^4^
D. Levofloxacin – Incorrect. Although fluroquinolones are frequently used for treatment of community-acquired pneumonia in high-risk patients due to their broad-spectrum of activity, they do not have anti-fungal activity.
E. Micafungin – Incorrect. Although echinocandins are frequently used for invasive fungal infections such as aspergillosis and candidiasis, they have limited data for efficacy for disseminated blastomycosis and inconsistent *in vitro* activity against *B. dermatitidis.*^5^

## Conflicts of interest

None disclosed.
